# Bioabsorbable fixation of a pediatric trochlear notch chondral injury

**DOI:** 10.1093/jscr/rjaf714

**Published:** 2025-09-05

**Authors:** Nicholas Frappa, Danil Chernov, Matthew G Alben, Samuel I Fuller, Sean Martin, Jeremy P Doak

**Affiliations:** Department of Orthopaedics, Jacobs School of Medicine and Biomedical Sciences, University at Buffalo, 955 Main Street, Buffalo, NY 14203, United States; Department of Orthopaedics, Jacobs School of Medicine and Biomedical Sciences, University at Buffalo, 955 Main Street, Buffalo, NY 14203, United States; Department of Orthopaedics and Sports Medicine, University at Buffalo, 462 Grider Street, Buffalo, NY 14215, United States; Department of Orthopaedics and Sports Medicine, University at Buffalo, 462 Grider Street, Buffalo, NY 14215, United States; Department of Orthopaedics and Sports Medicine, University at Buffalo, 462 Grider Street, Buffalo, NY 14215, United States; Department of Orthopaedics and Sports Medicine, University at Buffalo, 462 Grider Street, Buffalo, NY 14215, United States

**Keywords:** bioabsorbable fixation, trochlear notch, olecranon, fracture, chondral shear, pediatric, elbow injury

## Abstract

An 8-year-old girl fell onto her outstretched arm, sustaining proximal ulna and radial neck fractures. After closed reduction and casting in the emergency department, radiographs showed improved alignment but limited bony detail. A CT scan performed 3 days later demonstrated 18° apex-medial angulation of the radial neck, slight radiocapitellar subluxation, and subtle calcification near the trochlear notch, concerning intra-articular injury. Surgical exploration revealed a large cartilaginous shear fracture of the trochlear notch involving the coronoid. The fragment was anatomically reduced and fixed with bioabsorbable chondral fixation darts, and the olecranon was stabilized using Kirschner wires and a tension-band construct. Early follow-up demonstrated a functional range of motion without pain. Chondral shear injuries of the trochlear notch in children are rare lesions. Early recognition and fixation can restore joint congruity and function. Bioabsorbable implants offer a viable solution, even when the subchondral bone is minimal.

## Introduction

Chondral shear fractures of the pediatric elbow are exceedingly rare and pose diagnostic and treatment challenges. In young children, a large portion of the elbow joint is unossified, making radiographic diagnosis of intra-articular injuries difficult [1, 2]. These injuries are referred to as “TRASH” (The Radiographic Appearance Seemed Harmless) lesions, describing occult cartilage-based or osteochondral injuries not visible on plain films [1, 2]. The elbow comprises six ossification centers that fuse throughout development, complicating interpretation during skeletal immaturity [3]. When injury occurs before ossification is complete, purely chondral fragments may form, escaping detection on standard imaging [1, 2].

Capitellar fractures represent <1% of pediatric elbow fractures, typically affecting older children near skeletal maturity [2]. Equivalent shear injuries involving the ulnar side, specifically of the trochlear notch of the olecranon, are even less common [4, 5]. Olecranon fractures themselves are rare in children, most often affecting those aged 5–10 and frequently associated with radial neck fractures or Monteggia lesions [3, 6, 7]. In high-energy trauma, shear forces across the ulnohumeral joint can create displaced cartilaginous fragments, leading to joint incongruity and early arthritis if not promptly identified [2, 4]. Subtle findings such as joint effusion or loss of congruity should prompt advanced imaging [2].

We present a rare case of a pediatric shear-type chondral injury of the trochlear notch, treated operatively. We describe the use of bioabsorbable chondral fixation darts and place this case in the context of the evolving literature on cartilage-dominant elbow injuries. The patient and her parents were informed that data concerning the case data would be submitted for publication, and they provided consent.

## Case report

An 8-year-old right-hand-dominant female with a body mass index of 16.7 kg/m^2^ and no significant medical history presented after falling from monkey bars. She reported right elbow pain and inability to fully extend the joint. Examination revealed swelling, posterior tenderness, and limited range of motion.

**Figure 1:**
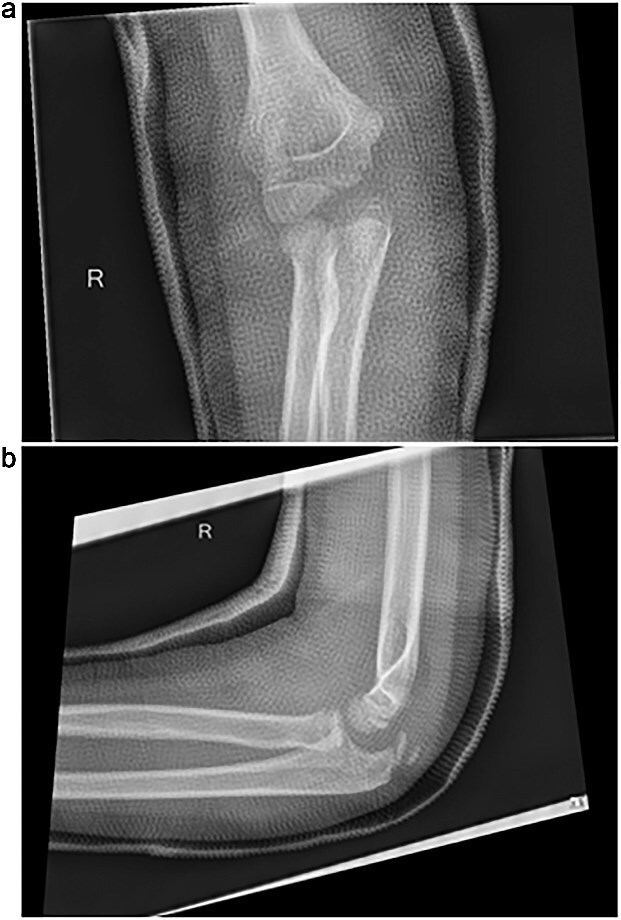
(a, b) Initial radiographs after closed reduction, showing proximal ulna and radial neck fractures.

**Figure 2:**
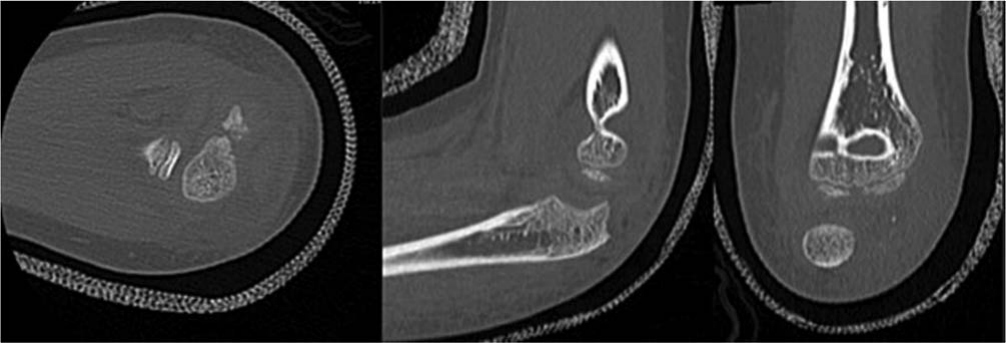
CT scan demonstrating radial neck angulation, radiocapitellar subluxation, and linear calcification near the trochlear notch.

Initial radiographs following closed reduction and casting demonstrated fractures of the proximal ulna and radial neck, with improved alignment but limited bony detail (Fig. 1a and b). Elbow effusion was also noted. A CT scan performed 3 days later showed 18° apex-medial angulation of the radial neck fracture, slight inferior subluxation of the radial head, and linear calcification near the ulnotrochlear interval, concerning for an intra-articular fragment (Fig. 2).

**Figure 3:**
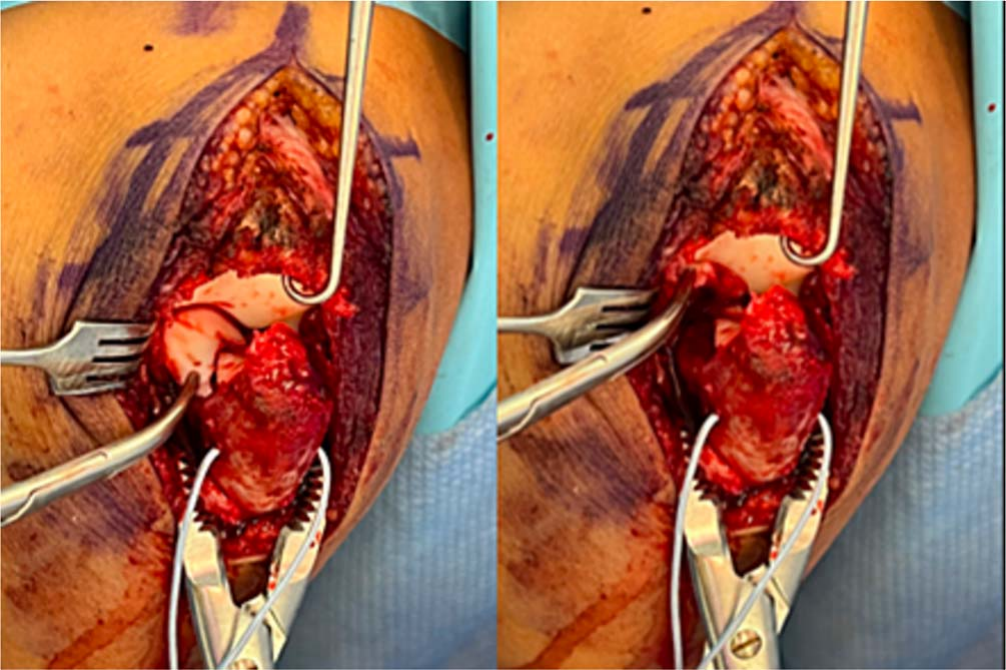
Intraoperative photographs showing a large chondral shear fragment involving the trochlear notch, visualized prior to reduction.

Surgical fixation was performed through a posterior approach. A large cartilaginous fragment involving the trochlear notch and coronoid articular surface was identified, lacking substantial subchondral bone (Fig. 3). The fragment was anatomically reduced using a mosquito clamp and fixed with three bioabsorbable chondral fixation darts. The olecranon fracture was stabilized with two Kirschner wires and a tension-band construct. Intraoperative fluoroscopy confirmed satisfactory alignment. The elbow was immobilized in a long arm cast in slight extension.

**Figure 4:**
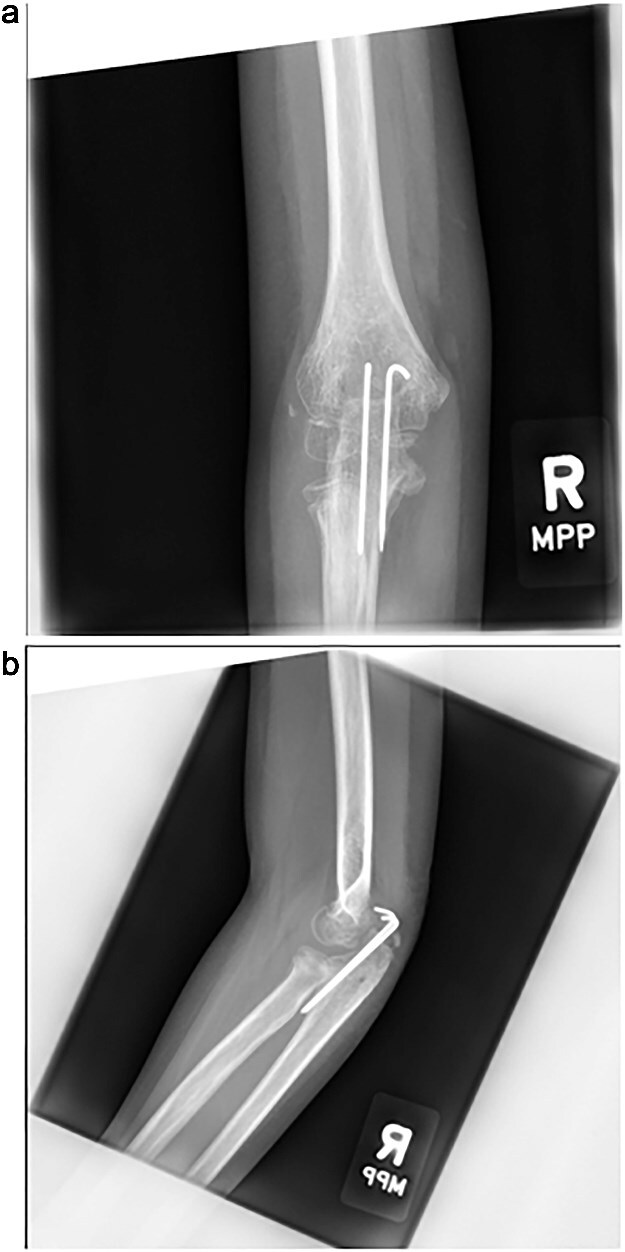
(a, b) Radiographs at 5 weeks, showing maintained alignment at the time of cast removal.

**Figure 5:**
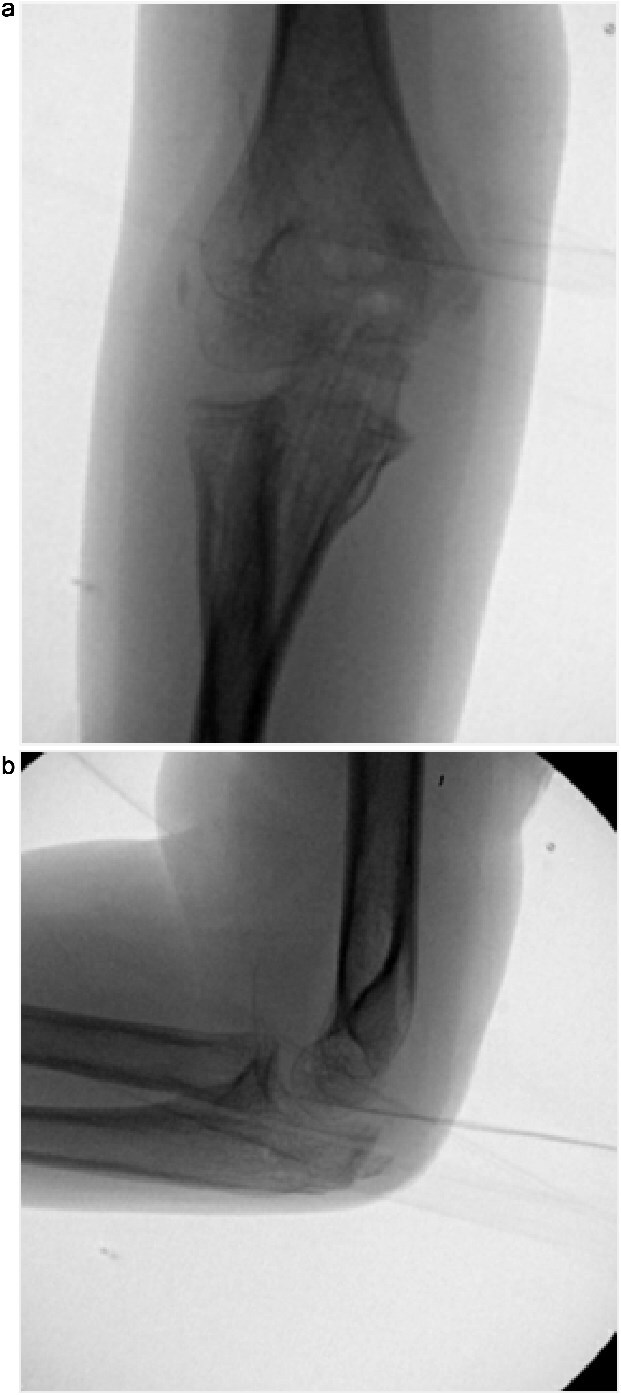
(a, b) Intraoperative fluoroscopic images following hardware removal at 4 months, demonstrating restoration of joint congruity.

The cast was removed at 5 weeks, and radiographs confirmed maintained alignment (Fig. 4a and b), allowing the patient to transition to a hinged elbow brace. Four months after surgery, she underwent planned removal of the Kirschner wires and tension band (Fig. 5a and b). At 6 months, elbow range of motion was 10°–130° on the right, compared to 0°–140° on the left, and she reported no pain with activities. She was subsequently lost to follow-up.

## Discussion

This case illustrates a rare pediatric elbow TRASH lesion: a predominantly chondral shear fracture of the trochlear notch. Diagnostic challenges are heightened in younger patients, where incomplete ossification renders cartilage-based injuries radiographically occult [1–3]. Although supracondylar, lateral condyle, and medial epicondyle fractures account for most pediatric elbow injuries, awareness of cartilage-dominant patterns remains essential [3].

The injury likely resulted from a shear force across the ulnohumeral joint during a fall onto an outstretched arm, causing a transient elbow dislocation that spontaneously reduced. Wang and Wang [4] described comparable cases, attributing coronoid osteochondral flap fractures to shear forces during spontaneous reduction. This mechanism may explain the linear calcification and radial head subluxation observed in our case. Because plain radiographs often fail to detect cartilage injuries, surgical exposure proved essential for definitive diagnosis and treatment.

Operative fixation presented unique challenges. The fragment clearly contributed to joint surface congruity but contained minimal bone. Given the patient’s young age and healing potential, bioabsorbable chondral fixation darts were used successfully. Although limited, the existing literature supports absorbable fixation in similar settings. Tsukamoto *et al.* [8] reported excellent outcomes using biodegradable pins for pediatric trochlear fractures. Kajiwara *et al.* [9] achieved healing with poly-l-lactic acid (pins. Kim *et al.* [10] and Valisena *et al.* [5] used absorbable sutures for coronoid cartilage flaps, while You *et al.* [11] and Pilotto *et al.* [12] demonstrated safe outcomes with bioabsorbable screw fixation for capitellum fractures. Collectively, these cases demonstrate that absorbable fixation can offer effective treatment options for pediatric cartilage injuries of the elbow, even when bony support is minimal or absent.

Our case adds to this growing evidence describing the use of bioabsorbable chondral fixation darts in a pediatric trochlear notch injury. Early stabilization preserved native joint anatomy and allowed functional recovery despite minimal subchondral bone. While long-term outcomes remain unknown, early intervention may reduce the risk of joint incongruity and degenerative changes. Continued reporting of rare pediatric cartilage injuries will be essential to refining diagnostic and treatment strategies and improving long-term outcomes in skeletally immature patients.
